# Willingness and hesitancy of parents to vaccinate against COVID-19 their children ages 6 months to 4 years with frail conditions in Italy

**DOI:** 10.3389/fpubh.2023.1212652

**Published:** 2023-07-13

**Authors:** Grazia Miraglia del Giudice, Giorgia Della Polla, Mario Postiglione, Italo Francesco Angelillo

**Affiliations:** ^1^Department of Experimental Medicine, University of Campania “Luigi Vanvitelli”, Naples, Italy; ^2^Department of Public Health and Laboratory Services, Teaching Hospital of the University of Campania “Luigi Vanvitelli”, Naples, Italy

**Keywords:** COVID-19, frail children, hesitancy, Italy, vaccination

## Abstract

**Background:**

In Italy, on December 2022, COVID-19 vaccination was recommended for children aged 6 months-4 years with frail conditions and for those healthy. The purposes of the survey were to understand parental willingness and hesitancy toward COVID-19 vaccination of children with frail conditions in Italy and related influencing factors.

**Methods:**

A cross-sectional survey was performed among 445 parents with a child aged 6 months-4 years with frail conditions who attended a teaching hospital and a public hospital randomly selected in the city of Naples, Italy.

**Results:**

Almost one third (29.9%) were willing to vaccinate their frail children against COVID-19, whereas 21.3% were uncertain, and 48.8% did not intend to vaccinate. Parents with a higher level of perception that the vaccine is useful and safe and those who had received information by pediatrician were more likely to be willing to vaccinate their child. The mean Parent Attitudes About Childhood Vaccines (PACV-5) score was 3.4, with 13.5% of parents high-hesitant for the COVID-19 vaccination for their child. Parents with a higher COVID-19 vaccine-related safety concerns, those who have delayed at least one shot of a recommended vaccine for their child, and those who did not have received at least three doses of the vaccine against SARS-CoV-2 were more likely to be high-hesitant.

**Conclusion:**

The survey findings have important implications for designing interventions to increase willingness and to reduce hesitancy for COVID-19 vaccine among parents of frail children aged 6 months-4 years in Italy.

## 1. Introduction

The novel coronavirus disease 2019 (COVID-19), caused by severe acute respiratory syndrome coronavirus 2 (SARS-CoV-2), determined over 767 million confirmed cases and 6 million deaths worldwide, whereas in Italy the total number of cases was more than 25.7 million and over 190 thousand people died (1). It is interesting to observe that the previous SARS-CoV-1 epidemic caused more than 8 thousand cases and 774 deaths worldwide (2), while in Italy only 4 non-fatal cases were reported (3).

It is well-known that COVID-19 vaccination has been the most important measure to mitigate the spread of the disease. The use of the mRNA COVID-19 vaccines in children ages 6 months to 4 years has been recommended by the European Medicines Agency on October 19, 2022. In children from 6 months to 4 years of age, Comirnaty can be given as primary vaccination with three doses (of 3 micrograms each) and the first two doses are given 3 weeks apart, followed by a third dose at least after 8 weeks, whereas Spikevax with two doses (of 25 micrograms each), 4 weeks apart (4). In Italy, on December 9, 2022, the Ministry of Health recommended the vaccination with Comirnaty for children with high frail conditions and for those healthy (5). However, to date, no vaccination campaign among this age group has been implemented. This is a public health concern as of May 3, 2023, among the 26 million confirmed individuals infected by SARS-CoV-2 in Italy, 785 thousand cases have been reported among children ages 6 months to 4 years and there have been over 14 thousand hospitalizations (6).

These children, mainly those with frail conditions, are at increased risk of SARS-CoV-2 infection and this is an important cause of morbidity and mortality (7–9). So, is even more important in them compared to the healthy population, to achieve an adequate coverage of the COVID-19 vaccination in order to reduce the burden of the disease and to control the transmission. There is overwhelming evidence supporting that parents are the decision-makers regarding the vaccination for their children and the attitudes are key determinants in the vaccine acceptance and uptake. Cross-sectional studies have investigated the willingness of parents of children ages 6 months to 4 years in many places (10–20) but limited information was available regarding children with frail conditions (21–23). Such data are paramount to COVID-19 vaccine uptake rates. Therefore, to address these gaps in the literature, the two purposes of the present survey were to understand the willingness and hesitancy of parents toward COVID-19 vaccination of their children ages 6 months to 4 years with frail conditions in Italy and to identify the influencing factors.

## 2. Materials and methods

### 2.1. Setting and participants

This cross-sectional survey was conducted between February and April, 2023, as part of a larger project, which aimed to investigate COVID-19 vaccination perceptions and behaviors of different groups in Southern Italy (24–33). The source population was all parents who had a child aged between 6 months to 4 years with frail conditions who attended a teaching hospital and a public hospital randomly selected in the city of Naples, Southern part of Italy, from January 1, 2023 to April 5, 2023.

The sample size was determined by using a single proportion formula by assuming the prevalence of parents’ willingness of COVID-19 vaccination for their children of 50%, with a 95% confidence interval, a 5% margin of error, and then adding a 10% non-response rate (34). Thus, the minimum total number of participants was 427.

### 2.2. Procedures

The parent of each child was contacted by trained investigators via telephone from Monday to Friday in morning, afternoon, and evening, to ensure that either working or not working parents were reached, or was approached while waiting for their child’s clinical appointment. At the beginning of the interview, the research team illustrated at the respondent parent the survey objectives and their rights as research participants, that the time commitment for completion of the survey was 10 min, that the participation was voluntary, that no subject identifiers were recorded, that the data would be maintained anonymously, and that they may freely stop answering the questionnaire at any point. If the parents had more than one frail child within this age range, they were requested to respond for the child whose age was closest to 6 months. All parents gave verbal informed consent prior to the interview. Participants received no incentives whatsoever for completing the survey. A maximum of 3 attempts were made to contact the parents via telephone. If the individual selected was unreachable he was not replaced.

The study protocol and procedures were approved by the Ethics Committee of the Teaching Hospital of the University of Campania “Luigi Vanvitelli” (protocol number 0001816/i).

### 2.3. Survey instrument

Data were collected using a structured questionnaire which was modified and adapted from those used by some of us enrolling different populations (24–33). A pilot study was conducted with 10 participants to assess the survey’s comprehensibility and feasibility of the questionnaire. Since, no modifications were made, the parents were included in the final sample.

The questionnaire, uploaded as Supplementary material, entailed the following three major sections: (1) socio-demographic and general characteristics of the respondent (i.e., gender, age, partnership status, education background, employment status, history of chronic medical condition, personal and family history of having been infected by SARS-CoV-2, and personal and family history of COVID-19 vaccination) and of the child (i.e., gender, age, birth order, and chronic medical condition); (2) attitudes toward the COVID-19 infection (perceived severity of COVID-19, perceived risk for the child of being infected by SARS-CoV-2) and the COVID-19 vaccine (perceived utility and safety). For these four items the responses were with a 10-point Likert scale with a score ranging from “1: Not at all” to “10: At all”, and for two items, whether they delayed or refused at least one shot of vaccinations for their children, with “yes/no/do not know” responses. Participants were also asked their willingness to vaccinate their child and the response options were “yes”, “no”, and “uncertain” and they were then asked to select from a provided list with 12 options of response one or more reasons for their willingness or unwillingness or uncertain to vaccinate their child. Participants’ vaccine hesitancy was measured based on the 5-item version of the 15-item Parent Attitudes About Childhood Vaccines (PACV) Survey Tool, with 5-point Likert categorical responses (strongly disagree, disagree, uncertain, agree, or strongly agree) (30, 35). Each item received a score of 0 for “non-hesitant” responses, a score of 1 for responses of “not sure” and “I do not know,” and a score of 2 for “hesitant” responses. The scores were summed to a total score ranging from 0 to 10 and each parent was considered low hesitant with a score of 0–4, moderate hesitant with a score of 5–6, and high hesitant with a score of 7–10; and (3) the last section is about the relevant information source(s) about this vaccination for their child and the need to receive additional information. In the question regarding the source(s) of information, the respondents were asked to select, from a list of 7 possible options, all sources that have been used. Finally, the response regarding the need of additional information was in the yes/no format.

### 2.4. Statistical analysis

First, descriptive statistics were expressed as frequencies, proportions, means, and standard deviations to assess the characteristics of the sample and of the different variables. Second, bivariate analysis with chi-square test or Student’s t-test were used to examine the association between categorical or continuous variables. Third, two multivariate logistic regression models were estimated to examine the extent to which independent variables, having in the bivariate analysis a *p*-value equal to or less than 0.25, were associated with the outcomes of interest. The models were built using a stepwise variable selection procedure, with a *p* = 0.2 for a variable to stay and a *p* = 0.4 to exclude it. The two outcomes of interest were the following: parental willingness to vaccinate against COVID-19 their child (Model 1); and parental COVID-19 vaccine high hesitancy for their child (Model 2). For Model 1, the response options were combined into “not willing/uncertain” and “willing”, to form a dichotomous outcome; for Model 2, the response options were combined into a dichotomous outcome “low hesitant with a score of 0–4 and moderate hesitant with a score of 5–6” and “high hesitant with a score of 7–10”. The independent variables included in the models were the following: gender (male = 0; female = 1), age, in years (continuous), partnership status (unmarried = 0; married/living with a partner = 1), at least one parent having baccalaureate/graduate degree (no = 0; yes = 1), at least one parent being a healthcare worker (no = 0; yes = 1), at least one parent/family member with one chronic medical condition (no = 0; yes = 1), having had a personal or family member history of SARS-CoV-2 infection (no = 0; yes = 1), having received at least three doses of the vaccine against SARS-CoV-2 (no = 0; yes = 1), at least one child who had received at least two doses of the vaccine against SARS-CoV-2 (no = 0; yes = 1), and having more than one child (no = 0; yes = 1). The following were the independent variables regarding the frail child: believing that COVID-19 is a severe illness (continuous), risk perception of getting SARS-CoV-2 infection (continuous), perceived utility of the COVID-19 vaccine (continuous), perceived safety of the COVID-19 vaccine (continuous), having delayed at least one shot of vaccine (no = 0; yes = 1), source of information about the COVID-19 vaccine (none = 1; pediatrician = 2; other = 3), age, in years (<1 = 1; 1 = 2; 2 = 3; 3 = 4; 4 = 5), gender (male = 0; female = 1), and having been infected by SARS-CoV-2 (no = 0; yes = 1).

The results of the logistic regression models were presented as odds ratios (OR) with 95% confidence intervals (CI). All hypothesis testing used two-tailed *p-*value equal to or less than 0.05 to be considered statistically significant. STATA software version 17 was used to conduct all statistical analyses.

## 3. Results

Of the 476 parents contacted, a total of 445 completed the interview with an effective response rate of 93.4%. The principal characteristics of the parent and of the child are provided in Table 1. The mean age of respondents was 35.4 years, the majority were female (86.1%) and married or living with a partner (93.7%), less than one-third had a university education (30.6%), half were employed (52.6%), less than one-fifth of the parents and of the other family members had at least one chronic medical condition, 82.7% have had a personal or family member history of SARS-CoV-2 infection, and 42.7% had received at least three doses of the vaccine against SARS-CoV-2 some time prior to the survey. Most of the frail children were female, the most frequent causes of frailty were prematurity (29.2%), kidney (26.5%), and cardiovascular diseases (22.9%), and almost half had been infected by SARS-CoV-2.

**Table 1:** Socio-demographic and general characteristics of the study population.

Characteristics	*N*	%
*Parent*		
Age, years	35.4 ± 5.6 (20–57)*	
Gender		
Female	383	86.1
Male	62	13.9
Partnership status		
Married/living with a partner	417	93.7
Unmarried	28	6.3
Educational level		
High school degree or less	305	69.4
Baccalaureate/graduate degree	136	30.6
Employment status		
Unemployed	211	47.4
Employed	234	52.6
At least one parent being a healthcare worker		
No	417	93.7
Yes	28	6.3
At least one parent/family member with one chronic medical condition		
No	361	81.1
Yes	84	18.9
Having had a personal/family member history of SARS-CoV-2 infection		
No	77	17.3
Yes	368	82.7
Having received at least three doses of the vaccine against SARS-CoV-2		
No	255	57.3
Yes	190	42.7
Having more than one child		
No	246	55.3
Yes	199	44.7
At least one child who had received at least two doses of the vaccine against SARS-CoV-2		
No	171	85.9
Yes	28	14.1
*Child*		
Age, years		
<1	44	10.1
1	121	27.8
2	96	22
3	76	17.4
4	99	22.7
Gender		
Female	239	54.6
Male	199	45.4
Frailty condition**		
Prematurity (<2 years of age)	130	29.2
Kidney	118	26.5
Cardiovascular	102	22.9
Metabolic	29	6.5
Onco-hematologic	25	5.6
Rheumatic	23	5.2
Genetic syndromes	25	2.9
Others (obesity, neurological)	12	2.7
Having been infected by SARS-CoV-2		
No	229	51.5
Yes	216	48.5

Number for each item may not add up to total number of study population due to missing value.

*Mean ± Standard deviation (range).

**More than one chronic medical condition was allowed to be indicated.

The results of the attitudes toward COVID-19 and its vaccination, measured on a 10-point Likert type scale, among parents who responded to the questionnaire are showed in Table 2. Only 19.5% of the sample perceived that their children were at risk of being infected by SARS-CoV-2 with an overall mean value of 6.6. Similar attitude has been observed regarding their perception that COVID-19 was a serious illness with only 16.4% indicated the value of 10 and the overall mean value was 6.5. Regarding the vaccination, respectively 13.9% and 12.4% of the respondents indicated that was useful to vaccinate their children and that the vaccine was safe with overall mean values of 4.8 and 5.6.

**Table 2:** Respondents’ attitudes toward COVID-19 and its vaccination measured on a 10-point Likert type scale.

Item	Mean ± SD*	Score of 1 *N* (%)	Score of 10 *N* (%)
How serious do you consider COVID-19 for your child?	6.5 **±** 2.5	16 (3.6)	73 (16.4)
How much do you perceive your child at risk of getting COVID-19?	6.6 **±** 2.6	20 (4.5)	87 (19.5)
How useful do you consider COVID-19 vaccination for your child?	4.8 **±** 3.3	129 (29.1)	62 (13.9)
How safe do you consider COVID-19 vaccination for your child?	5.6 **±** 2.9	78 (17.5)	55 (12.4)

*Standard deviation.

Overall, almost one third (29.9%) of the participants responded that they were willing to vaccinate their frail children against COVID-19, whereas 21.3% were uncertain, and 48.8% did not intend to vaccinate. Table 3 presented the multivariate logistic regression analysis of the factors affecting each of the different outcomes of interest. Eight variables were incorporated into the final model regarding the parental willingness to vaccinate their child against COVID-19 and four of them were found to be significantly associated with the outcome. These included parents’ perceived utility and safety of the vaccination and source of information. Respondents who had a higher level of perception that the vaccine is useful (OR = 2.58; 95% CI = 1.99–3.34) and safe (OR = 1.66; 95% CI = 1.27–2.17) and those who had received information by pediatrician, with the odds of being willing 83% higher as compared to those who had received information from other sources (OR = 0.17; 95% CI = 0.04–0.75) and 68% higher compared to those who did not receive any (OR = 0.32; 95% CI = 0.11–0.99), were more likely to be willing to vaccinate their child (Model 1). The most prevalent reasons among the parents who said that they would vaccinate against COVID-19 their frail children were to protect them (55.6%), the vaccines’ efficacy (48.1%), and the child is at risk of getting a COVID-19 infection (46.6%). The main reasons why parents were uncertain to vaccinate their children were the concern over side effects of the COVID-19 vaccine (63.1%) and not having received a pediatrician’s recommendation (26.3%), whereas the main reasons for their unwillingness were the concern for the side effects (75.6%) and that the child is not at risk of getting a COVID-19 infection (31.8%).

**Table 3:** Multivariate logistic regression analysis results examining the determinants of the different outcomes of interest.

Variable	OR	SE	95% CI	*p*
*Model 1*. Parental willingness to vaccinate their child against COVID-19 Log likelihood = −86.17, *χ*^2^ = 56.53 (8 df), *p* < 0.0001				
Higher perceived utility of the vaccine against SARS-CoV-2	2.58	0.34	1.99–3.34	<0.001
Higher perceived safety of the vaccine against SARS-CoV-2	1.66	1.23	1.27–2.17	<0.001
Source of information about the vaccine against SARS-CoV-2 for their child				
Pediatrician	1.00°			
Other sources	0.17	0.13	0.04–0.75	0.02
None	0.32	0.18	0.11–0.99	0.049
Child’s age, years				
<1	1.00°			
1	0.59	0.31	0.21–1.67	0.326
3	0.36	0.24	0.11–1.31	0.123
4	0.59	0.31	0.21–1.64	0.312
Not having had a personal or family member history of SARS-CoV-2 infection	0.59	0.31	0.21–1.65	0.322
*Model 2*. Parental COVID-19 vaccine high hesitancy for their child Log likelihood = −126.59, *χ*^2^ = 86.12 (4 df), *p* < 0.0001				
Lower perceived safety of the vaccine against SARS-CoV-2	0.63	0.04	0.56–0.72	<0.001
Having delayed at least one shot of a recommended vaccine for their child	2.33	0.99	1.01–5.36	0.047
Not having received at least three doses of the vaccine against SARS-CoV-2	0.49	0.17	0.25–0.99	0.05
Male child	0.69	0.23	0.36–1.32	0.267

°Reference category.

The mean PACV-5 score was 3.4 with 13.5% of participating parents classified as high-hesitant for the COVID-19 vaccination for their child with a score ≥ 7, 14.2% as moderate-hesitant scoring between 5 and 6, and 72.3% as low-hesitant scoring ≤4. Responses to the individual items of the PACV-5 are shown in Table 4. Only 11.3% of parents thought their child received more vaccines than are good for them, more than one-fourth (28.6%) wanted children to receive fewer vaccines at the same time and more than three-quarters (80.8%) strongly disagreed or disagreed that it is better for children to develop immunity by getting sick than to get a shot. Approximately two-thirds (63.5%) of respondents considered themselves to be vaccine hesitant. Less than half (41.6%) said they trusted the information they received about childhood COVID-19 vaccine. The results of the multivariable logistic regression analysis examining the factors associated with the hesitancy showed that parents with a higher COVID-19 vaccine-related safety concerns (OR = 0.63; 95% CI = 0.56–0.72), those who have delayed at least one shot of a recommended vaccine for their child (OR = 2.33; 95% CI = 1.01–5.36), and parents who did not have received at least three doses of the vaccine against SARS-CoV-2 (OR = 0.49; 95% CI = 0.25–0.99) were more likely to be high-hesitant (Model 2 in Table 3).

**Table 4:** Descriptive characteristics of PACV-5 about COVID-19 vaccine.

Item	Parent response	*N* (%)
Children get more shots than are good for them	Strongly agree/agree	50 (11.3)
	Strongly disagree/disagree	369 (83.1)
	Not sure	25 (5.6)
It is better for my child to develop immunity by getting sick than to get a shot	Strongly agree/agree	43 (9.7)
	Strongly disagree/disagree	359 (80.8)
	Not sure	42 (9.5)
It is better for children to get fewer shots at the same time	Strongly agree/agree	127 (28.6)
	Strongly disagree/disagree	281 (63.3)
	Not sure	36 (8.1)
Overall, how hesitant about the COVID-19-vaccine for your child would you consider yourself to be?	Very hesitant/ somewhat hesitant	282 (63.5)
	Not hesitant at all/not too hesitant	12 (2.7)
	Not sure	150 (33.8)
I trust the information I receive about the COVID-19-vaccine	Strongly agree/agree	185 (41.6)
	Strongly disagree/disagree	118 (26.6)
	Not sure	141 (31.8)

Number for each item may not add up to total number of study population due to missing value.

Only one-fourth of parents (26%) reported having previously received information on the COVID-19 vaccine for their child. Of those who had acquired information, the pediatrician was indicated as the principal source by 57.7%. The second most reported source was Internet (31.9%), followed by friends and family members (28.5%) and mass media (22.4%). Finally, 44.3% participants indicated the desire to get additional information about the COVID-19 vaccine.

## 4. Discussion

This survey, to the best of our knowledge the first conducted in Italy, provided important and useful insights into the parents’ willingness and hesitancy to have their children ages 6 months to 4 years with frail conditions vaccinated against COVID-19 as well as the influencing factors.

The first key finding is that of the parents surveyed, only 29.9% said that were willing to vaccinate their children against COVID-19 and this is of great concern although no data are available on the effectiveness and safety of vaccines in this age group. This result was like that of 31.3% in the United States among parents of healthy children 2–4 years (15). This value was higher than those observed in other countries, in which the prevalence was 19.8 and 25.2% among parents of healthy children, respectively, aged <5 years (20) and 0–4 years (16) in the United States, but it was lower than the 71% in Brazil (14) and 50.6% in Ireland for children aged 0–4 years (23), 50% for 4 years in Australia (19), 45.1% for <5 years with few had an underlying disease in Malaysia (13), 42% for <2 years in the United States (12), and 41.9 and 45.4% for 2–4 years and 6–23 months in Canada (17). Furthermore, the value was also likewise lower as compared to the 36% found among parents of children 0 to 5 years with developmental disabilities in the United States (22), and to the 42.1% of children 0 to 4 years with neurodevelopmental disorders in Bangladesh (21). However, it is necessary to underline that the variations in reported prevalence across countries may in part be attributed to the differences in, for example, the frequency of the disease, study setting and period, characteristics of the sample, and data collection methodology. Moreover, among the parents who participated in this survey, 13.5% reported being high-hesitant assessed using the PACV-5 questionnaire against the COVID-19 vaccine for their frail children, whereas the moderate-hesitant and low-hesitant accounted, respectively, for 14.2% and 72.3% of the total participants. In a study among parents of healthy children aged 0–60 months in Turkey it has been observed that 9.38% of participants were vaccine hesitant measured using the PACV-15 (36). It is important to underline that the sample of this survey was constituted by children with chronic medical conditions and this may partially explain the parents’ hesitancy, possibly due to the presence of individuals for whom vaccines are contraindicated (37, 38).

The second key finding is that identifying the primary reasons for parents’ willingness or unwillingness of the COVID-19 vaccination for their child are needed by the healthcare workers, mainly pediatricians with whom they have a closer and deeper interaction, to tailor information. More than half of the surveyed parents answered that the prevention of the onset of the disease was the main reason taken into consideration for their willingness in favor of the COVID-19 vaccination for their child, consistent with the existing literature among parents of children of different age (30, 39, 40). The fact that the parents have indicated this reason underlined the stone that the disease severity is a leverage point to get their children vaccinated. Among those parents who were uncertain or did not intend to vaccinate their child, the most common reason was the parental concerns about the safety of the vaccination. This result is in accordance with those that have been observed in other recent studies worldwide examining immunization confidence among different groups of individuals (14, 22, 41–44). Therefore, it is of great importance targeting the interventions to address these concerns raised by the parents by a participatory approach and it is also necessary to stress the dangers of this vaccine-preventable disease.

The third key finding was, unexpectedly and unfortunately, a widespread lack of information among the sample of this study with only slightly more than one-third of the parents had acquired information about the COVID-19 vaccination for their children through multiple sources. The most frequently chosen sources of information were pediatricians and Internet. It is worth mentioning that the information from pediatricians is crucial and it was significantly associated with the parents’ willingness to vaccinate against COVID-19 their children. Among parents who have sought information about this vaccination for their child from this source the odds of being willing to vaccinate were 83% higher as compared to those who had received information from other sources and 68% higher compared to those who did not receive any. This result support previous publications in the literature which underline the significant and positive influence of pediatricians and other healthcare professionals on the individuals’ attitudes and behaviors with those who had received information from this source that were more likely to accept or to receive a vaccination than those who did not use this source (11, 13, 30, 45, 46). However, since slightly more than half had received information by their pediatricians about the COVID-19 vaccine this may represents a limitation to receive the vaccine in the future. This indicates the need for specific strategies and actions to ensure healthcare professionals communication and education programs to inform and to persuade parents of frail children to get the vaccine against COVID-19 and also to impart knowledge about its benefits in order to change their behavioral intentions. Moreover, although the amount of health-related information seeking online is continuing increasing and Internet is a very common used platform, the fact that 31.9% used this source is of concern because previous studies have reported the availability of widespread misinformation with the vaccines on SARS-CoV-2 that are considered unsafe and harmful and this is likely to negatively influence parents’ knowledge and to reduce the intentions to vaccinate their child (46–48). Therefore, public health campaigns regarding the benefits of this vaccination on this source should be more frequent so that the misinformation is less likely to be seen and, as such, will have a less negative impact.

The fourth key finding was that in the final multivariate logistic regression models, several factors significantly predicted the parents’ willingness and hesitancy toward the vaccination against SARS-CoV-2 for their child. It is important to note that, in addition with the association already reported with the source of information, respondent’s attitudes have been identified as important determinants. Indeed, this study revealed that willingness to vaccinate their child against COVID-19 is heavily influenced by parental positive attitude toward the utility and safety of this vaccination. Parents who had these positive attitudes were 2.58 and 1.66 times more willing to vaccinate their child when compared with parents who had unfavorable attitudes. This finding is consistent with those of several recently conducted studies showing that parents’ opinions about the efficacy of the vaccines in general have a significant impact in vaccine acceptance (13, 15, 18, 23, 49, 50). Therefore, public health education programs for parents with a negative attitude toward the utility of the COVID-19 vaccine would result in a high vaccine acceptance with an increase in the uptake. The survey revealed that parents with a lower COVID-19 vaccine-related safety concerns had 37% lower odds of being high-hesitant than those with higher concern and those who delayed at least one shot of a recommended vaccine for their child were 2.33 times more likely to be high-hesitant. This finding is consistent with those observed in previously conducted surveys (49, 51). Moreover, delayed of at least one shot of a recommended vaccine for their child underlined the need of targeted activities through a better parent-physician communication that may be crucial toward enhancing vaccination coverage.

## 5. Limitations

This survey is subject to at least five potential methodological limitations that merit consideration when interpreting the present findings. First, the survey adopted a cross-sectional design and this limits to make final determinations about the causal relationships between the independent variables and the outcomes of interest. Second, the data collection source was a single-site and therefore generalization of the study findings to other geographic locations of Italy and globally should be done with caution. Third, no record verification was performed regarding the vaccination status. However, the parental interview is the most used method and recall bias is likely limited as the survey was conducted when no active vaccination campaign among this age group has been implemented. Fourth, responses are prone to social desirability bias in which parents tend to give answers that are thought to be more socially acceptable and may not correlate with future behaviors, such as the intention to vaccinate their children. However, this bias has been minimized by assuring all participants that their responses were anonymous and confidential. Fifth, it is possible that non-respondents were different than respondents. Non-response bias was minimized by attempting to reach parents at least 3 times over the telephone and it can be ruled out since a participation rate of 93.4% has been obtained. Despite the described limitations, the findings of this survey give important information of the parents’ willingness and hesitancy toward the COVID-19 vaccination of their frail children ages 6 months to 4 years in Italy.

## 6. Conclusion

In conclusion, the survey findings have important implications for designing interventions to increase willingness and to reduce hesitancy for COVID-19 vaccine among parents of frail children ages 6 months to 4 years in Italy. Healthcare providers, mainly pediatricians, have a significant role and closer and regular contacts with the parents should be encouraged for increasing awareness about the importance of vaccinating their child and acceptance of COVID-19 vaccination. Furthermore, the finding that less than 60% of the parents have received the recommendation by a pediatrician emphasizes that improving their training with also informative campaigns is essential to assist the parents by communicating with them helpfully and to support them in order to have the correct information regarding the COVID-19 vaccine’s safety and utility for reaching a higher coverage.

## Data availability statement

The raw data supporting the conclusions of this article will be made available by the authors, without undue reservation.

## Ethics statement

The study protocol and procedures were approved by the Ethics Committee of the Teaching Hospital of the University of Campania “Luigi Vanvitelli” (protocol number 0001816/i). All parents gave verbal informed consent prior to the interview.

## Author contributions

GMdG, GDP, and MP participated in the conception and design of the study, collected the data, and contributed to data analysis and interpretation. IFA, the principal investigator, designed the study, was responsible for the statistical analysis and interpretation, and wrote the article. All authors have read and approved the final version of the article and agreed to be accountable for all aspects of the work.

## Funding

This work was supported by a grant of the Regione Campania (DGR n. 140/2020 Urgent measures regarding the containment and management of the epidemiological emergency from COVID-19, POR Campania FESR 2014–2020, Asse Prioritario 1 “Ricerca e Innovazione”).

## Acknowledgments

The authors would like to extend their gratitude to all parents who participated by completing the questionnaire.

## Conflict of interest

The authors declare that the research was conducted in the absence of any commercial or financial relationships that could be construed as a potential conflict of interest.

## Publisher’s note

All claims expressed in this article are solely those of the authors and do not necessarily represent those of their affiliated organizations, or those of the publisher, the editors and the reviewers. Any product that may be evaluated in this article, or claim that may be made by its manufacturer, is not guaranteed or endorsed by the publisher.
